# Response to: ‘Antenatal depression and the association of intimate partner violence among a culturally diverse population in southeastern Norway’ by Melby et al.

**DOI:** 10.18332/ejm/157996

**Published:** 2023-02-14

**Authors:** Heru S. W. Nugroho, Sri Winarni, Koekoeh Hardjito, Suparji Suparji, Nani Surtinah, Bringiwatty Batbual, Wiwin Martiningsih

**Affiliations:** 1 Department of Midwifery, Poltekkes Kemenkes Surabaya, Surabaya, Indonesia; 2 Department of Nursing, Poltekkes Kemenkes Malang, Malang, Indonesia; 3 Department of Midwifery, Poltekkes Kemenkes Malang, Malang, Indonesia; 4 Department of Midwifery, Poltekkes Kemenkes Kupang, Kupang, Indonesia

**Keywords:** antenatal depression, intimate partner violence, individual characteristics

**Dear Editor,**

We have carefully reviewed an article that discusses antenatal depression. In this case, antenatal depression is significantly influenced by intimate partner violence (any lifetime-IPV, fear, emotional-IPV, physical-IPV and sexual-IPV). Meanwhile, of the eleven individual characteristic variables, six variables affect antenatal depression, namely civil status, family income, pregnancy planned, tobacco, alcohol and alcohol partners; while five variables (age, education level, occupation, mother tongue, and parity) did not affect it^1^.

There are three questions related to this condition: 1) ‘Is it true that age, education level, occupation, mother tongue and parity do not affect antenatal depression?’; 2) ‘Could the five variables influence antenatal depression indirectly?’; and 3) ‘What statistical analysis can be used to analyze this indirect effect?’.

In this study, the researchers used regression analysis, so that in the hypothesis it is assumed that all individual characteristic variables simultaneously affect antenatal depression directly. Referring to relevant references, these eleven variables should not all affect antenatal depression directly. There are several variables that influence antenatal depression indirectly through intermediary variables, for example: parity affects pregnancy planned, then pregnancy planned affects antenatal depression.

Referring to a similar case^2^, we present an alternative framework for the relationship between related variables (Figure 1), which should be reviewed and revised before use. In this case, logistic regression analysis cannot be used, and the most appropriate choice is path-analysis^3,4^. Because the researchers use categorical data, one of the statistical programs that can be used is Smart-PLS^3^.

**Figure 1:**
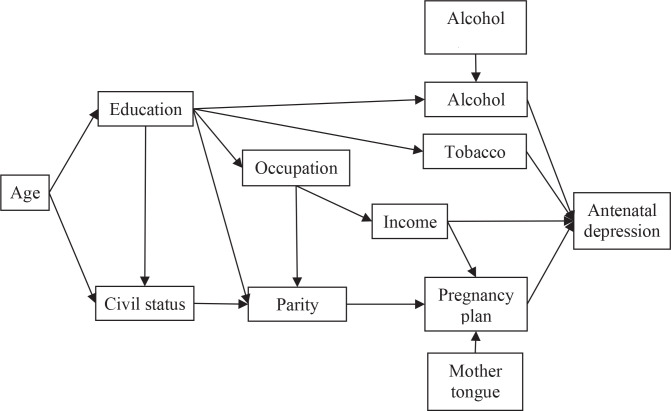
Pathways of influence of individual characteristic factors related to antenatal depression

Furthermore, we suggest that researchers perform further analysis using path-analysis, to obtain more complete information about the effect of these eleven variables on antenatal depression.

## Data Availability

Data sharing is not applicable to this article as no new data were created.
